# In a Rat Model of Acute Liver Failure, Icaritin Improved the Therapeutic Effect of Mesenchymal Stem Cells by Activation of the Hepatocyte Growth Factor/c-Met Pathway

**DOI:** 10.1155/2019/4253846

**Published:** 2019-11-07

**Authors:** Lu Wang, Shu Li, Han-Yu Wang, Juan Zeng, Zheng-Zheng Zhang, Dong-Yong Lv, Wei-Hong Kuang

**Affiliations:** ^1^The First Affiliated Hospital of Guangzhou University of Chinese Medicine, Guangzhou, Guangdong 510405, China; ^2^Guangzhou University of Chinese Medicine, Guangzhou, Guangdong 510405, China; ^3^The Research Center of Basic Integrative Medicine, Guangzhou University of Chinese Medicine, Guangzhou, Guangdong 510006, China; ^4^Lingnan Medical Research Center of Guangzhou University of Chinese Medicine, Guangzhou, Guangdong 510405, China

## Abstract

Acute liver failure (ALF) is a serious life-threatening condition. Mesenchymal stem cells (MSCs) may be an effective treatment for this condition and a good alternative to liver transplantation. Icaritin (ICT) is an active ingredient of the genus* Epimedium*, a traditional Chinese medicine, with the potential to enhance the proliferation of MSCs. The purpose of this study was to explore whether ICT increased the therapeutic effects of MSCs and explore its underlying mechanisms. For* in vivo *experiments, a rat ALF model was established by intraperitoneal injection of D(+)-galactosamine/ lipopolysaccharide. MSCs cocultured with ICT were used to treat ALF rats and the protective effects assessed as survival rate, levels of serum AST and ALT, and histological changes in liver tissue. For* in vitro* experiments, MSCs were treated in serum-free culture for 72 h to simulate the disruption of intrahepatic microcirculation. MSCs apoptosis was examined to determine whether ICT rescued impaired MSCs. The role of the hepatocyte growth factor (HGF)/c-Met pathway in MSCs was assessed by constructing genetically modified MSCs overexpressing c-Met and by using the c-Met receptor inhibitor (crizotinib). The results showed that MSCs increased the survival rate of ALF rats and reduced liver damage. MSCs cocultured with ICT exerted a greater therapeutic effect than MSCs alone. Further, the HGF/c-Met pathway played a key role in the antiapoptotic activity of MSCs, which was associated with the optimized efficacy of ICT. In conclusion, this study demonstrated that ICT enhances the therapeutic effect of MSCs in a model of ALF, improving the antiapoptotic potential of MSCs by upregulation of the HGF/c-Met pathway. The combination of stem cell therapy with traditional herbal extracts may improve MSC-based clinical applications.

## 1. Introduction

Acute liver failure (ALF) is a severe, rapidly deteriorating disease characterized by the abrupt onset of severe liver injury and high mortality. Liver transplantation is one of the most effective treatment for ALF; however, the shortage of donor organizations remains a major obstacle to its application. Hence, alternative treatments are urgently needed.

Recently, mesenchymal stem cells (MSCs) have shown excellent potential for many therapeutic applications. A large number of studies based on stem cell transplantation have achieved remarkable results and provided new ways for the treatment of various diseases. For ALF, MSCs are a promising treatment because they have the potential to differentiate into hepatocytes to restore liver function and provide immune regulation to suppress inflammatory storms [1]. Many studies [2] have shown that MSCs transfusion is safe and feasible for liver failure; it can improve liver function and ascites in experimental animal and patients [3]. However, the efficacy of MSCs is limited by their poor survival rate and their inability to adapt to extreme pathological environments [4]. Therefore, researchers must increase the number of stem cell infusions and the frequency of infusion therapy, which greatly affects the efficacy and, on the other hand, increases the cost of treatment and reduces treatment compliance, making it difficult to popularize. Therefore, there is an urgent need for strategies to improve the efficacy of stem cell therapies.

Many therapeutic strategies have been devised to improve stem cell adaptability to unfavorable environments. Silk Fibroin Complex Matrices have functioned as ideal scaffold for MSCs [5]. A nanoparticle was fabricated and coated with red blood cell membranes that increased blood stability and carried useful factors from MSCs [6]. A combination of MSCs and nanoparticles, loaded with IL-lRa, was investigated [7]. In recent studies, reprogrammed pluripotent cell-specific factors (CXCR4 and Oct4) were used to construct gene-modified MSCs to promote colonization of MSCs [8–12], but the use of new materials such as nanoparticles or scaffolds is limited because of their high cost. Moreover, the carcinogenic risk of gene modification technology remains controversial. Soluble cytokine growth factors, such as platelet-derived growth factor (PDGF) and fibroblast growth factor (FGF), have been reported to promote the proliferation of MSCs, although the mechanism is unclear. Their use is limited because they are cost prohibitive and place cells in a state of stress [13]. Therefore, attention has turned to natural herbs and their vast milieu of phytochemicals as replacements for synthetic formulations [14]. As alternative therapeutics, natural products such as purified compounds from traditional herbal extracts have exceptional characteristics: low toxicity, affordability, and availability [13]. Studies evaluated the effects and mechanisms of herbal extracts on the biological activity of MSCs, including proliferation, differentiation, apoptosis, autophagy, and senescence [13, 15–17]. Icaritin (ICT) is a hydrolytic product of icariin derived from the genus* Epimedium*, which is a traditional Chinese herbal medicine, broadly used for a range of diseases such as growth retardation. Previous studies confirmed that ICT promotes the proliferation of MSCs [18–20]. Our previous data have shown that ICT can promote proliferation of MSCs and increase mRNA expression of a variety of cytokines including hepatocyte growth factor (HGF), urokinase plasminogen activator (uPA), matrix metalloproteinase-1 (MMP-1), and vascular endothelial growth factor (VEGF) [20]. In this study, we evaluated the therapeutic efficacy of MSCs cultured with ICT in ALF rats. Further, the underlying mechanistic basis for the impact of ICT on MSCs was assessed.

## 2. Materials and Methods

### 2.1. Materials and Reagents

The following instruments and reagents were used: icaritin (ICT, purity *⩾* 98%; Sigma, St. Louis, MO, USA); galactosamine (D-GalN; Sigma); lipopolysaccharide (LPS; Sigma); human HGF (ReproTech, Columbia, Missouri, USA); crizotinib (MedChemExpress, Monmouth Junction, NJ, USA); monoclonal rabbit anti-human/rat antibody for cleaved caspase-3 (Cell Signaling Technology, Boston, MA, USA; no. #9664); monoclonal mouse anti-human/rat antibody for Bcl-2 (Abcam, Cambridge, MA, USA; no. ab692); monoclonal rabbit anti-human/rat antibody for Bax (Abcam; no. ab32503); monoclonal mouse anti-human/rat antibody for c-Met (ThermoFisher, Waltham, MA, USA; no. k845.5); monoclonal rabbit anti-human/rat antibodies for glyceraldehyde-3-phosphate dehydrogenase (GAPDH, Abcam; no. ab181602); goat anti-rabbit IgG labeled with horseradish peroxidase (HRP; Abcam; no. ab6721) and goat anti-mice IgG labeled with HRP (Abcam; no. ab6789); and bicinchoninic acid (BCA) protein assay kit (FD, Dalian, China).

### 2.2. Cell Isolation and Culture

Alliancells Institute of Stem Cells and Translational Regenerative Medicine provides the human umbilical cord-MSCs (hUCMSCs) we use. Identification of hUCMSCs was shown in Supplementary Materials (Figures [Supplementary-material supplementary-material-1] and [Supplementary-material supplementary-material-1]). MSCs from umbilical cord were isolated and expanded according to the method of Hanyu Wang which was described in the previous literature [21, 22]. Briefly, the umbilical cord was firstly cut into pieces (1-3mm^3^), and then it was digested with collagenase II (Gibco, Carlsbad, CA, USA) for 1h. The mixture was passed through a 100-*μ*m filter, so cell suspensions were obtained. Then cells were washed with PBS twice and cultured in culture medium which contained Dulbecco's Modified Eagle Medium (DMEM)/F12 (Gibco, USA), 10% fetal bovine serum (FBS) (Biological Industries, Cromwell, CT, USA), 1% glutamine (Sigma Aldrich), and 1% penicillin-streptomycin (Gibco, USA). MSCs at passages 3-6 were used for experiments unless otherwise stated. For* in vitro* experiments, MSCs were incubated with ICT (0.1 *μ*M, Sigma), recombinant HGF (25ng/ml, Peprotech), or crizotinib (4*μ*M, MedChemExpress) according to the requirement of the experiment [23, 24].

### 2.3. Animal Preparation and Experimental Groups

The animal experiments were performed in the Experimental Animal Laboratory of the First Affiliated Hospital of Guangzhou University of Chinese Medicine (Guangdong, China) (approval no. SYXK [Yue] 2013–0092). All experimental procedures were approved by the Ethic Committee of the First Affiliated Hospital of Guangzhou University of Chinese Medicine (approval no. TCMF1-2016030). A total of 85 specific-pathogen-free (SPF) adult SD rats (7-10 weeks old; 300-350 g) were purchased from the Medical Experimental Animal Center of Guangdong Province (Guangdong, China). The rats were individually placed in a ventilated breeding room and subjected to 12 hours of light and dark light cycles under controlled humidity and temperature conditions. They have sterile water and sterile standard particle rodent feed. All rats were anesthetized with chloral hydrate to relieve pain. All efforts were made to reduce suffering. The rats were randomly allocated into four groups: normal group (nonmodel), NS group (normal saline treatment), MSCs treatment group, and ICT + MSCs (ICT precultured MSCs) group. The acute liver failure model was established by intraperitoneal injection of D-(+)-galactosamine (D-GalN) 0.8g/kg in combination with lipopolysaccharide (LPS) 5 *μ*g/kg. Liver damage and failure were manifested in encephalopathy, diarrhea, ascites, and other symptoms, with a dramatic rise in the concentration of transaminases. The criterion for successful ALF rat models is the histopathological massive necrosis of hepatocytes and the 48-hour mortality rate is between 50 and 100%. In the preexperimental stage, the above modeling scheme can reach this criterion. The NS group was intraperitoneally injected with 1 ml of normal saline 1 h after ALF model establishment. The MSCs group was intraperitoneally injected with 1 ml of a MSCs cell suspension (approximately 5×10^6^/ml) 1 h after model establishment. For the ICT + MSCs group, 1 ml of MSCs suspension (approximately 5×10^6^/ml) precultured with 0.1 *μ*M ICT for 3 days was intraperitoneally injected 1 h after model establishment (the cells were washed twice with saline before they were injected). All rats were observed at 12 h, 24 h, 36 h, and 48 h after model establishment. Rat survival was observed at the designated time point. Blood of surviving rats was collected from the abdominal aorta at 24 h and 48 h, and liver tissue (cooled in liquid nitrogen and stored at -80°C) collected for further analysis.

### 2.4. Biochemical Analysis of Liver Function

The blood was centrifuged for 15 minutes at 4°C for 1000 g to obtain serum. Serum alanine aminotransferase (ALT) and aspartate aminotransferase (AST) levels were measured by automatic biochemical analyzer.

### 2.5. Cell Viability Was Assessed by the MTS Method

Cells were seeded at a density of 5 × 10^4^ cells/ml in a 96-well plate (100ul per hole). The detection of cell viability was performed following the instruction of the CellTiter 96® AQueous Nonradioactive Cell Proliferation Assay (Promega, Corporation, Madison, WI, USA). Absorbance at 490 nm was measured using an EnSpire Multimode Plate Reader (PerkinElmer, Shelton, CT, USA). All samples are evaluated in triplicate.

### 2.6. Hematoxylin and Eosin Staining of Liver Tissue

The collected liver tissues were immediately cut into pieces, fixed in a 4% paraformaldehyde solution for 24 h, embedded in paraffin, then cut into 4 *μ*m slices, stained with hematoxylin and eosin, and observed with an optical microscope (Motic, USA).

### 2.7. Western Blotting Analysis

The collected liver tissues from different groups were lysed in radioimmunoprecipitation assay (RIPA) lysis buffer to extract total proteins. Total protein concentration was determined using the BCA Protein Assay Kit. Then the proteins were loaded and separated by SDS-PAGE (sodium dodecyl sulphate-polyacrylamide gel electrophoresis) and then transferred to polyvinylidene fluoride membranes (Millipore). After soaking in blocking liquid for 2 h, polyvinylidene fluoride membranes were transferred to primary antibodies solution and incubated at 4°C overnight to react with Bcl-2 (1:500), cleaved caspase-3 (1:500), Bax (1:1000), and GAPDH (1:2000), respectively. Then the membranes were washed with Tris-buffered saline with Tween-20 (TBST) three times, transferred to secondary antibodies (1:4000) solution for 2 h at room temperature. They were washed with TBST again, and then Luminata Crescendo Western HRP Substrate (Bio Rad, Berkeley, CA, USA) was added. The membranes were exposed in a gel imaging analysis system (Alpha Innotech FluorChem FC2, USA). Images were analyzed using ImageJ software to determine gray scale. Samples were assessed in triplicate using different batches samples.

### 2.8. Enzyme-Linked Immunosorbent Assay (ELISA)

MSCs were cultured with ICT at different concentrations for 72 h. HGF in the cultural supernatant was quantified by ELISA kit (R&D System, USA), according to the protocol provided by the manufacturer.

### 2.9. Quantitative Real-Time Reverse Transcriptase Polymerase Chain Reaction (qRT-PCR)

Total RNA was isolated from fresh cells using an Eastep® Super Total RNA Extraction Kit (Promega, USA). First-strand cDNA was synthesized using a Transcriptor First-Strand cDNA Synthesis Kit (Roche, Vaud, Switzerland). qRT-PCR was performed using FastStart Universal SYBR Green Master Mix (Roche). Each cDNA reaction was prepared from 1 *μ*g RNA and diluted to 20 *μ*l of the final volume, 2 *μ*l cDNA was subsequently used for each PCR reaction, and the reaction mixture had a total volume of 20 *μ*l containing 10 *μ*l SYBR Green Master(2x), 1*μ*l forward primer, 1 *μ*l reverse primer, 2 *μ*l cDNA, and 6 *μ*l H2O. The PCR conditions were as follows: 95°C for 10 min for preincubation; 95°C for 10 sec and 60°C for 30 sec for amplification; 65°C for 10 sec and 95°C for 10 sec to melting curve. The relative level of gene expression was normalized to an internal control (level of 18S rRNA) and calculated using the 2^-ΔΔ^CT method. The primer sequences were human c-Met sense primer sequence: 5′ TGCAAGGGAGAAGACTCCTA -3′ and antisense primer sequence: 5′- TATCCGGGACACCAGTTCA-3′; human Bax sense primer sequence: 5′ GATGCGTCCACCAAGAAGCT -3′ and antisense primer sequence: 5′- CGGCCCCAGTTGAAGTTG -3′; human Bcl-2 sense primer sequence: 5′ TCATGTGTGTGGAGAGCGTC -3′ and antisense primer sequence: 5′- AGCCTCCGTTATCCTGGATC -3′; human caspase 3 sense primer sequence: 5′ CTCCACAGCACCTGGTTATT -3′ and antisense primer sequence: 5′- AAGCTTGTCGGCATACTGTT -3′; human 18S rRNA sense primer sequence: 5′- CCTGGATACCGCAGCTAGGA' and antisense primer sequence: 5′- GCGGCGCAATACGAATGCCCC -3′. Biological repeats were performed using three different samples for each genotype, and technical triplicates were carried out for each gene expression analysis.

### 2.10. Flow Cytometric Detection of Apoptosis by Annexin V-FITC/PI Dual-Staining

Annexin V-FITC/PI staining was used for the quantification of early and late apoptotic cells. Detection was performed with an Annexin V-FITC/PI Apoptosis Detection Kit (KeyGEN BioTECH, Nanjing, China). MSCs from each experimental group were stained with Annexin V- FITC (5 *μ*l) and PI (5 *μ*l) for 10 min and then examined by flow cytometry (FACS Calibur, BD Biosciences, San Jose, CA, USA). The experiments were done in triplicate using different batches samples.

### 2.11. DNA Transfection and Treatment

Plasmid was purchased from the Zhongyuan Company (86498 pT3-EF1aH c-Met; ADDGENE, Cambridge, MA, USA) and extracted using a high-purity plasmid miniprep kit (MGGE, Chattanooga, Tennessee, USA) according to the protocol provided by the manufacturer. Gene transfection was carried out according to a published protocol by the cationic liposome method [25]. Umbilical cord (UC)-MSCs were seeded into 6-well plates at a density of 1.0 × 105 cells per well in stem cell culture medium containing 10% FBS, gently washed twice with PBS, and allowed to grow until 50% to 60% confluent. Next, 1000 *μ*l OPTI-MEM (Invitrogen, USA) medium was added to each well and incubated at 37°C in a humidified atmosphere containing 5% CO2. Lipofectamine 2000 (Invitrogen, USA) (5 *μ*l) was diluted with OPTI-MEM medium to a final volume of 500 *μ*l, incubated for 5 min at room temperature; 2 *μ*g of plasmid containing the full-length sequence of c-Met was added to each well; and OPTI-MEM was then added in a total volume of 500 *μ*l for 5 min at room temperature. Finally, 1000 *μ*l of the transfection complex liquid was added to each well and shaken slightly. The cells were incubated at 37°C in a humidified atmosphere containing 5% CO2 for 5 h. The transfection complex was replaced with fresh medium containing 10% FBS. The cells were collected at 48 h and washed twice prior to western blot analysis. The success of transfection was assessed by western blot analysis and qRT-PCR.

### 2.12. Statistical Methods

SPSS 23.0 (USA) was used to process the experimental data as means ± standard deviation (SD). All data were checked for normality and homogeneity of variance. One-way ANOVA was used to compare different treatment groups for the normally distributed data. Survival analysis was performed by the Kaplan-Meier method. In all analyses,* P* < 0.05 was considered statistically significant.

## 3. Results

### 3.1. ICT Enhanced the Therapeutic Effect of MSCs in a Rat Model of Acute Liver Failure (ALF)

**Figure 1:**
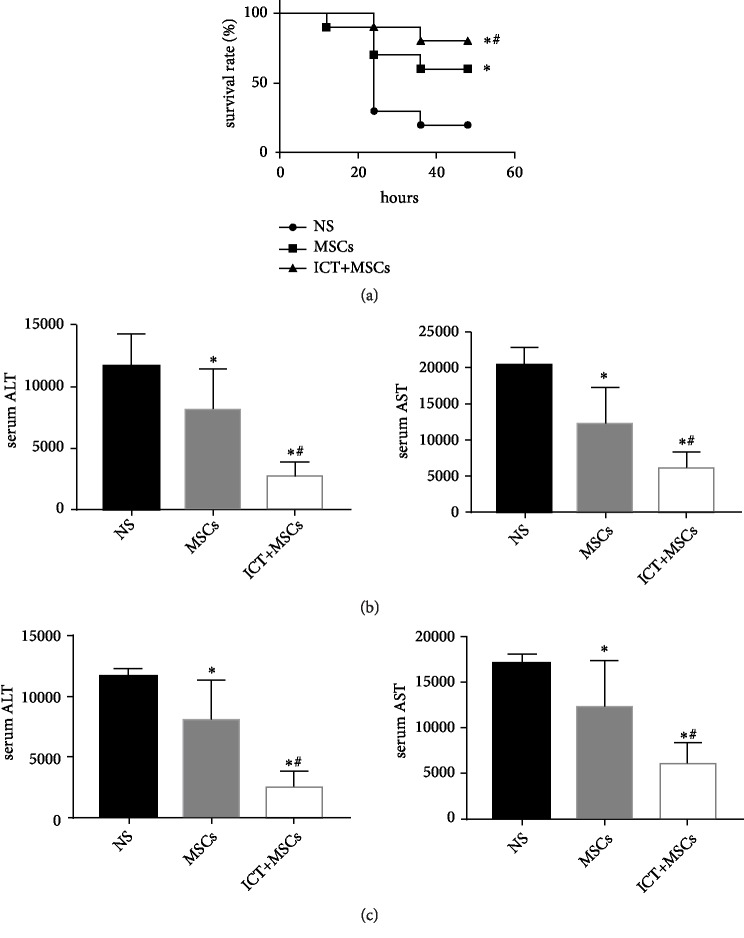
MSCs cocultured with ICT improved survival rates and hepatic function in a rat model of ALF. (a) Survival analysis of D-GalN/LPS-treated ALF rats in each group. (b) AST and ALT enzyme levels in peripheral blood samples collected at 24 h after model establishment. (c) AST and ALT enzyme levels in peripheral blood samples collected at 48 h after model establishment. ^∗^P < 0.05* vs. *the NS group; ^#^P < 0.05* vs. *the MSCs group. Data are means ± SD. Each experiment was replicated 6 times. Abbreviations. MSCs: mesenchymal stem cells; ALT: alanine aminotransferase; AST: aspartate aminotransferase.

ALF in rats was established by injection of D-GalN/LPS intraperitoneally. After 12h, the survival rate of NS group was 90%, while the survival rate of MSCs group and ICT+MSCs group was 90% and 100%, respectively. After 24h, the survival rate of NS group was 30%, while the survival rate of MSCs group and ICT+MSCs group was 70% and 90%, respectively. After 36h, the survival rate of NS group was 20%, while the survival rate of MSCs group and ICT+MSCs group was 70% and 90%, respectively. After 48h, the survival of NS group was 20%, while the survival rate of MSCs group and ICT+MSCs group was 60% and 80%. Compared with the NS group, the survival rate of the MSCs group and ICT+MSCs group was significantly improved (P<0.05). Compared with the MSCs group, the survival rate of the ICT+MSCs group was statistically increased (P<0.05) (Figure 1(a)). Serum biochemical index was evaluated at 24 h and 48 h. When compared to the MSCs group, rats receiving MSCs cocultured with ICT showed improved liver function. Compared to the NS group, liver function in both the ICT+MSCs and MSCs groups was significantly improved (Figures 1(b) and 1(c)).

### 3.2. ICT+MSCs and MSCs Improved Liver Pathology and Decreased Apoptosis of Hepatocytes

**Figure 2:**
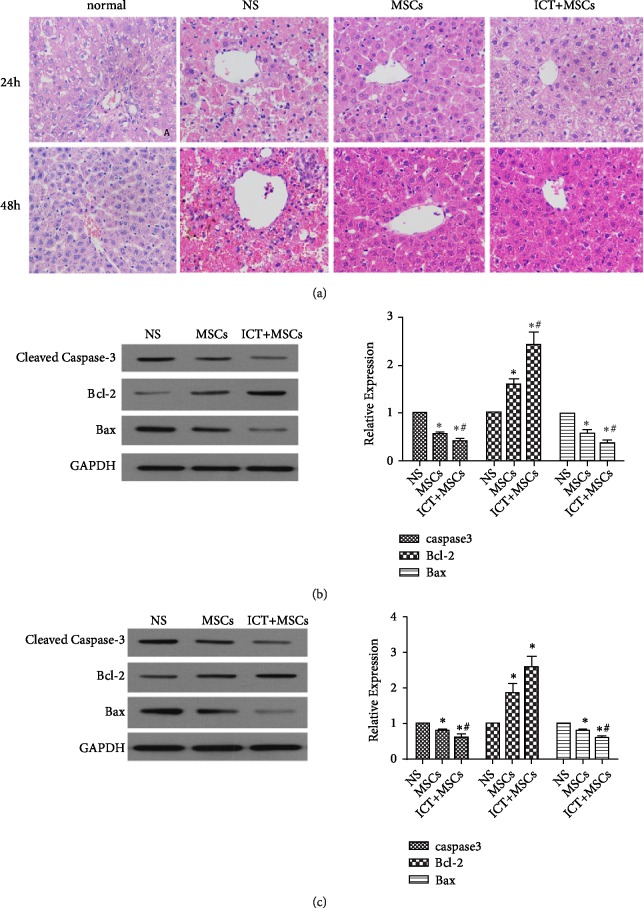
ICT+MSCs and MSCs improved liver histopathology and reduced the apoptosis of hepatocytes. (a) Hematoxylin and eosin staining of liver sections (400 ×). (b) qRT-PCR and western blot analysis of (cleaved) caspase 3, Bax, and Bcl-2 at 24h after induction of the ALF model. (c) qRT-PCR and western blot analysis of (cleaved) caspase 3, Bax, and Bcl-2 at 48 h after induction of the ALF model. ^∗^P < 0.05* vs. *NS group; ^#^P < 0.05* vs. *MSCs group. Data are means ± SD. Each experiment was replicated 6 times.

Hematoxylin and eosin staining was performed to assess the effect of MSCs on rat liver histology during ALF. Normal rats had uniform cellular morphology. The hepatocytes were arranged in an orderly manner and the structure of the liver lobules was clear. The hepatic cords were arranged radially with a central vein at the center. The liver tissue of rats in the NS group showed destruction of hepatic lobules, extensive hepatocyte necrosis with swollen cytoplasm, and the infiltration of inflammatory cells. Injured liver recovery was continuously investigated in the MSCs and ICT+MSCs groups. Liver tissue structure for the MSCs group had a slightly irregular arrangement, with infiltration of inflammatory cells around the central vein and the hepatic sinus. For the ICT+MSCs group, liver tissue structure was further improved, with hepatocytes arranged regularly and structurally. Slight edema and a small amount of inflammatory cell infiltration were observed. Hepatocytes morphology was improved for the MSCs and ICT+ MSCs groups. ICT+MSCs and MSCs inhibited hepatocyte inflammation and hepatic lobule destruction (Figure 2(a)).

Caspase 3 is the crucial executory enzyme of apoptosis, playing a vital role in the process of apoptosis. Bcl-2 is one of the essential antiapoptotic proteins of the Bcl family, while Bax is a proapoptotic protein. These proteins were assessed by western blot and RNA expression levels by qRT-PCR in liver tissue 24 h and 48 h after model establishment. As shown in Figure 2, compared to the NS group, treatment with MSCs and ICT+MSCs resulted in a significant increase in Bcl-2 and a decrease in caspase 3 and Bax, suggesting an inhibition of cellular apoptosis. Furthermore, the ICT+MSCs group exhibited a greater effect than MSCs, which suggests that ICT enhanced the antiapoptotic effect of MSCs on hepatocytes (Figures 2(b), 2(c), and [Supplementary-material supplementary-material-1]).

### 3.3. ICT Treatment Increased the Viability of MSCs during Hydrogen Peroxide-Induced Cytotoxicity

Initial experiments assessed the cytotoxic effects of various hydrogen peroxide concentrations on MSCs at various periods of exposure. By MTS assay, hydrogen peroxide significantly decreased cell viability at a concentration of 400 *μ*M for 6 h. This concentration and period of exposure were selected for subsequent experiments.

**Figure 3:**
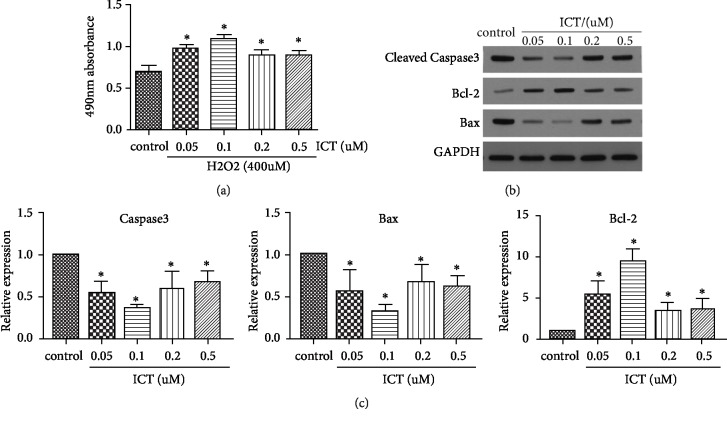
ICT protects MSCs from hydrogen peroxide-induced cell death and the expression of apoptosis-related proteins. (a) The effect of different concentrations of ICT on the viability of hydrogen peroxide treated MSCs. (b) Detection of protein levels of cleaved caspase-3, Bcl-2, and Bax in MSCs as judged by western blot. (c) Detection of mRNA expression levels of caspase-3, Bcl-2, and Bax in MSCs by qRT-PCR. ^∗^P < 0.05* vs. *control. Data are means ± SD. Each experiment was replicated 3 times.

MSCs were treated with various concentrations of ICT simultaneously in the setting of hydrogen peroxide. By MTS assay, reduced cell viability due to hydrogen peroxide was effectively reversed by pretreatment with ICT. Compared to control, ICT treatment increased viability of MSCs in concentrations dependent manner. Compared to 0.1 *μ*M, the viability of MSCs at 0.2 *μ*M and 0.5 *μ*M was lower (P < 0.05). These results demonstrate that ICT can reduce hydrogen peroxide-induced death of MSCs, with 0.1 *μ*M ICT being most effective. Therefore, 0.1 *μ*M ICT was selected as the optimum concentration for the subsequent experiments (Figure 3(a)).

ICT pretreatment not only significantly restored cell viability but also upregulated expression of BCL-2 and downregulated the expression of Bax and caspase 3. Consistent with the MTS results, the effect of 0.1 *μ*M ICT was more obvious than the other concentrations. These results suggest that ICT enhances the antiapoptotic activity of MSCs during oxidative stress in vitro (Figures 3(b) and 3(c)).

### 3.4. ICT Inhibits the Apoptosis of MSCs via the HGF/c-Met Pathway

**Figure 4:**
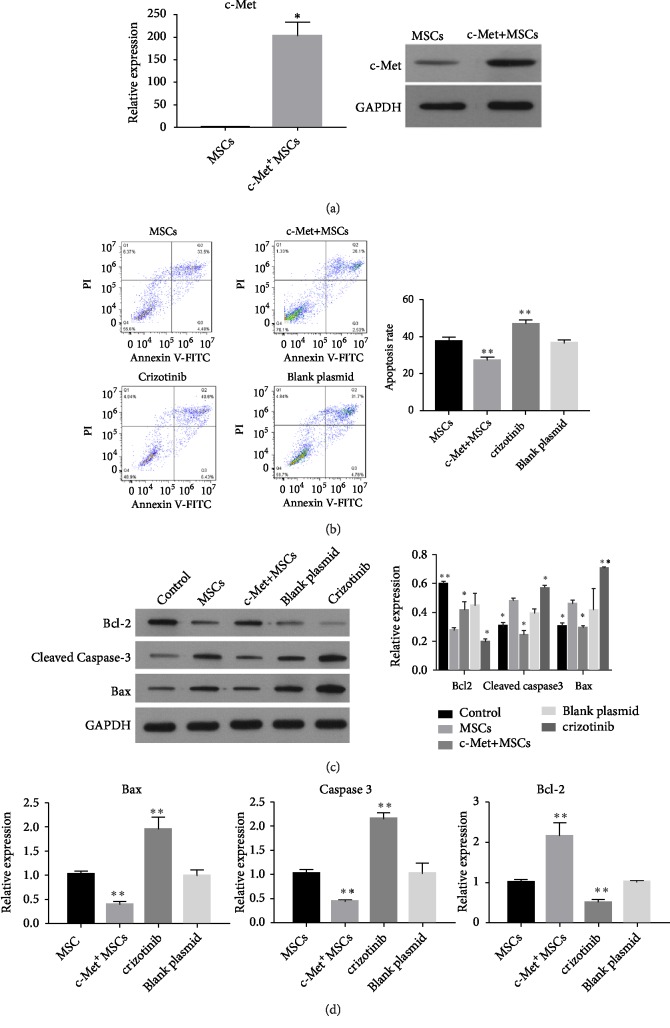
The antiapoptotic effect of the HGF/c-Met signing pathway in MSCs. (a) The expression of c-Met in genetically modified MSCs was examined to verify successful transfection. Left: c-Met gene relative expression by qRT-PCR. ^∗^P < 0.05* vs. *MSCs; right: c-Met expression assessed by western blot. (b) The percentage of apoptotic cells by Annexin V-FITC/PI staining and flow cytometric analysis. (c) Western blot analysis of cleaved caspase 3, Bax, and Bcl-2 expression after serum-free culture for 72 h. (d) mRNA expression levels of caspase-3, Bcl-2, and Bax in MSCs after serum-free culture for 72 h by qRT-PCR. ^∗^P < 0.05* vs. *MSCs. Each experiment was replicated 3 times.

UC-MSCs can secrete high levels of HGF and also express the HGF receptor, c-Met. It is possible that the antiapoptotic effect of MSCs may be via the HGF/c-Met pathway in an autocrine fashion. The molecular and cellular basis for this is not established. Hence, genetically modified MSCs were constructed to overexpress c-Met. c-Met overexpression was verified by qRT-PCR and western blot. The results of qRT-PCR demonstrated c-Met expression to be higher in c-Met+MSCs compared with the null cells (P < 0.05), which was confirmed by western blot, demonstrating that gene transfection was successful (Figure 4(a)).

As described before, disturbance of microcirculation is a key pathological mechanism of acute liver failure. Stem cells have a high apoptotic rate and poor survival in harsh microenvironments, which restricts their usefulness. Hence, hydrogen peroxide was used to simulate an oxidative stressful environment, and serum-free culture conditions were used to mimic disturbance of microcirculation during liver failure. Based on results of initial experiments, serum-free culture for 72 h was chosen for the subsequent experiments.

A c-Met receptor inhibitor (crizotinib) was used to assess the antiapoptotic effect of the HGF/c-Met pathway in MSCs. As shown in Figure 4(b), Annexin V/PI double staining was used to assess MSC apoptosis. FITC−/PI−, FITC+/PI−, FITC+/PI+, and FITC−/PI+ were identified as living, early apoptotic, late apoptotic, and necrotic cells, respectively. MSCs cultured for 72 h in serum-free conditions had a high level of apoptosis, while c-Met+MSCs had a noticeable decrease in apoptosis (from 37.53±2.14%to 27.34±1.64%). For MSCs treated with crizotinib, apoptosis was increased (from 37.53±2.14%to 47.14±2.00%). These results suggest the HGF/c-Met signing pathway to have an increased antiapoptotic effect on MSCs.

Crizotinib treatment was also used to assess expression levels of apoptosis-related proteins affected by the HGF/c-Met pathway. As expected, levels of Bcl-2 were significantly reduced and the expressions of cleaved caspase-3 and Bax were increased when MSCs were treated with crizotinib. For c-Met+MSCs, Bcl-2 expression was upregulated, and cleaved caspase-3 and Bax were downregulated. The blank plasmid had no effect on protein expression levels (Figures 4(c) and 4(d)).

**Figure 5:**
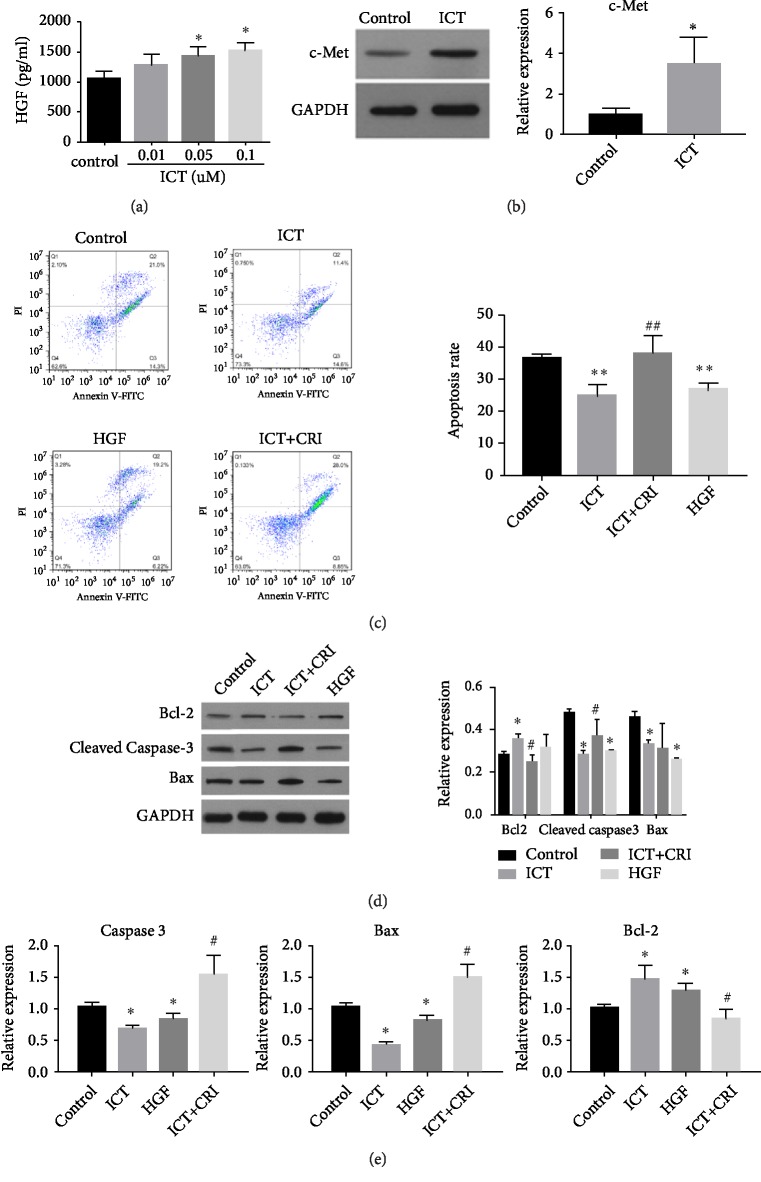
ICT decreases MSCs apoptosis via the HGF/c-Met pathway. (a) HGF levels for MSCs cultured with increasing concentrations of ICT as judged by ELISA. (b) Effect of ICT on MSCs of the c-Met receptor in vitro. Left: western blot for levels of c-Met. GAPDH served as a positive control. Right: mRNA levels of c-Met with various treatments. ^∗^P < 0.05 vs. control group. (c) Apoptosis was examined by Annexin V-FITC/PI staining and flow cytometric analysis. (d) Western blot analysis of cleaved caspase 3, Bax, and Bcl-2 levels. (e) mRNA expression levels of caspase-3, Bcl-2, and Bax in MSCs by qRT-PCR. ^∗^P < 0.05 vs. control group;^ #^P < 0.05 vs. ICT group. Each experiment was replicated 3 times.

MSCs were cocultured with ICT for 72 h to assess the effect of ICT on the HGF/c-Met signing pathway* in vitro*. HGF production by MSCs in the culture supernatants was detected by ELISA. ICT promoted MSCs secretion of HGF at concentrations of 0.01, 0.05, and 0.1*μ*M (P < 0.05),with 0.1*μ*M being the most effective (Figure 5(a)). It also increased production of the HGF receptor, c-Met. Western blot analysis demonstrated that ICT significantly increased levels of c-Met when compared to the control group, which was confirmed by qRT-PCR (P < 0.05) (Figure 5(b)).

These data demonstrated that ICT can increase the expression of HGF and c-Met. Annexin V/PI double staining was used to assess whether ICT could rescue impaired MSCs by reducing cellular apoptosis. For MSCs treated with ICT or HGF, the percentage of apoptotic cells was significantly reduced. The percentage of apoptotic cells was 24.73±3.54% (ICT group)* vs.* 26.68±2.13% (HGF group)* vs*. 36.55±1.28%(control group). The c-Met receptor inhibitor, crizotinib, reduced the antiapoptotic effect of ICT, 24.73±3.54% (ICT group) vs. 38.08±5.48%(ICT+ crizotinib group), suggesting that ICT inhibits serum-free culture induced apoptosis of MSCs (Figure 5(c)).

For analysis of apoptosis-related proteins, the results of western blot demonstrated that ICT and HGF can significantly increase the expression of Bcl-2 and decrease the expression of cleaved caspase-3 and Bax. Crizotinib reversed the effect of ICT, which was verified by qRT-PCR. These results indicate that ICT exerts an antiapoptotic effect in MSCs through regulation of the HGF/c-Met pathway (Figures 5(d) and 5(e)).

## 4. Discussion

Hepatic failure is characterized by a large number of dead or apoptotic hepatocytes, with a cascade of inflammatory cells as key pathological indicators. MSCs can differentiate into various cell types that can play important roles in tissue regeneration and repair. Further, MSCs can secrete multiple cytokines, such as VEGF-a, insulin like growth factor-1, epidermal growth factor, and keratinocyte growth factor [26]. MSCs have immunomodulatory properties by suppression of inflammatory responses through production of many different soluble cytokines [1, 27, 28]. MSCs are appropriate for autologous and allogeneic transplantation because they lack costimulatory molecules and HLA-II [29]. Human umbilical cord-MSCs (hUCMSCs) are a type of multipotent stem cell derived from umbilical cord Wharton jelly. Similar to bone marrow-derived MSCs (BMMSCs), they have a high self-replicative capacity and low immunogenicity. Additionally, they are more “young” and have unique advantages: the genes of hUCMSCs were closer to embryonic stem cells; they have stronger proliferation ability and differentiation potential; and they are convenient to take, noninvasive, with no ethical concerns, and easier to separate and have higher amplification efficiency. Therefore, hUCMSCs have higher utilization value and can be used for mass production [30]. Many published articles have demonstrated MSCs to be therapeutic in liver failure by secretion of trophic and immunomodulatory factors that support hepatocyte function, promote proliferation, inhibit apoptosis, promote angiogenesis, and reverse liver fibrosis before transdifferentiation [31]. They create a microenvironment for tissue regeneration while restraining life-threating cytokine storms and immunocyte infiltration. Furthermore, MSCs have the potential to treat liver failure in both experimental animals [5–7, 32, 33] and clinic patients [3, 34, 35]. Scholars have demonstrated that the MSCs transplantation is safe and feasible for the treatment of liver failure. The MSCs transfusions can significantly reduce mortality rate, lower the end-stage liver disease scores, increase serum albumin and cholinesterase, and increase prothrombin activity and platelet counts, while decreasing alanine aminotransferase levels and serum total bilirubin. Besides, the occurrence of short-term side effects and long-term complications was not significantly changed. However, therapeutic efficacy is hindered by the poor survival rate of stem cells, so researchers must increase the number of stem cell infusions and the frequency of infusion therapy; patients received the infusions of 1.0-10×10^5^ UC-MSCs per kilograms, and the MSCs were given once a week. However, the low survival rate of stem cells is attributed to an intolerance for adverse microenvironments. Therefore, the focus of ongoing research is to increase the tolerance of stem cells to adverse environments including ischemia, hypoxia, and oxidative stress.

Traditional Chinese herbal medicine is a treasure that has not been fully exploited. Modern pharmacological studies have identified and analyzed the herbal active ingredients and increased understanding of their value. The use of stem cells with herbal monomers is at the forefront of ongoing research. It remains obscure whether Chinese herbal extracts could improve the adaptability of stem cells to poor microenvironments. Based on traditional Chinese medicine theory, the kidney stores the congenital essence, in charge of human growth, development, reproduction, and aging. The stem cells have the characteristics of self-renewal and multidirectional differentiation, and they are the original cells that form the tissues of various organs. Stem cells participate in the same physiological process and have the same effect as the “innate essence” of the kidney. Stem cells may be the performance of “kidney essence” at the cellular level, which lays a theoretical foundation for the combination of traditional Chinese medicine and stem cell therapy. In recent years, many studies [13, 14] have shown that Chinese medicine for reinforcing kidney and their extract can promote replication and differentiation of embryonic stem cells, mesenchymal stem cells, hematopoietic stem cells, hair follicle stem cells, and other stem cells, further confirming the feasibility of Chinese medicine for tonifying the kidney combined with stem cell transplantation for disease treatment.

The genus* Epimedium*, belonging to Chinese medicine for reinforcing kidney, has many functions such as enhancing immunity, delaying aging, relieving osteoporosis, preventing cardiovascular and cerebrovascular diseases and reproductive system diseases, and inhibiting cancer. Epimedium has a variety of active ingredients, including icariin, icaritin, and polysaccharide. Icaritin (ICT) is one of the main active components with the molecular formula C_21_H_20_O_6_ and molecular weight of 368.38. As an extract of* Epimedium*, icaritin (ICT) may strengthen the capacity of stem cells. The studies found that ICT has many pharmacological and biological activities. ICT has anticancer effects for many types of cancer including human hepatoma cells [36, 37] and colon cancer cells [38]. It can increase the proliferation of mouse embryonic stem cells (mESCs) while maintaining their self-renewal capacity* in vitro *and pluripotency* in vivo* [18]. It also can enhances the proliferation, migration, and osteogenic differentiation of MSCs [19]. Therefore, we used MSCs pretreated with ICT to treat ALF rats and found that liver function, pathology, survival rate, and cell apoptosis were improved compared to MSCs alone (Figures 1 and 2). The significance of this discovery lies in the combination of traditional Chinese herbal medicine and modern medicine (e.g., stem cell therapy), providing new ideas and perspectives that may enhance clinical efficacy. A further aim of this study was to explore the underlying mechanisms of efficacy.

We found that treatment with ICT significantly increased HGF levels, promoting the expression of its receptor, c-Met (Figures 4(a) and 4(b)). HGF is a growth factor for various cells such as hepatocytes and epithelial cells with evidence demonstrating that HGF plays an important role in growth stimulation, migration, morphogenesis, angiogenesis, tissue regeneration, tumorigenesis, and tumor invasion [39]. Genetic modifications have verified the critical role of c-Met in liver regeneration and repair, while disruption of c-Met deceased hepatocyte survival rate and impeded organizational restructuring. Those studies suggest that the HGF/c-Met signaling pathway is required for the recovery of an injured liver [40]. Recent evidence indicates that HGF downregulates apoptosis signaling and the oxidative stress reaction in endothelial cells (ECs) [41, 42] and smooth muscle cells [43]. HGF/c-Met may exert these effects through both PI3-kinase/Akt and MAPK pathways [44]. HGF also protects hepatocytes against Fas-mediated apoptosis [45]. Recent studies have shown that UC-MSCs express high levels of HGF, with the expression of UC-MSCs being 30 times higher than that of BMMSCs [46]. However, when MSCs are differentiated into MSCs-derived-hepatocyte-like cells (HLCs), HGF expression was decreased dramatically and accompanied by impaired immunosuppressive function and diminished therapeutic effect [22]. C-Met, also known as hepatocyte growth factor receptor (HGFR), is encoded by the c-Met protooncogene, which is a crucial regulator of invasive growth that is expressed by both stem and cancer cells. HGF is activated when its cognate receptor, c-Met, is ligated [47]. In this study, we investigated whether c-Met+MSCs enhanced the antiapoptotic capacity of MSCs in serum-free culture. Results showed c-Met+MSCs significantly increase the number of viable cells and decrease the rate of apoptosis, while the c-Met inhibitor, crizotinib, weakened the antiapoptotic effect (Figure 4), indicating that the HGF/c-Met signing pathway plays a key role in protecting stem cells against adverse circumstances such as ischemia. Further, we observed the effects of ICT on apoptosis and the relationship with HGF/c-Met pathway. Findings revealed that ICT markedly suppressed the apoptosis of MSCs in serum-free culture, HGF exerted similar antiapoptotic effects, and crizotinib eliminated the protective impact of ICT on MSCs, suggesting that the efficacy of ICT upon MSCs was closely related to the HGF/c-Met pathway.

There are some limitations in this study which we would like to point out to improve future investigations. This study was designed with insufficient breadth and depth. For example, there is no genetic or proteomic analysis of ICT to understand macroscopically the regulation of stem cell transcription and translation by ICT. In addition, the up- and downregulation mechanism of ICT inhibiting stem cell apoptosis through HGF/c-Met pathway have not been carried out.

In conclusion, our findings confirm the therapeutic potential of ICT when combined with MSCs and provide a strong rationale for future clinical studies. The molecular and cellular mechanistic basis for these optimized therapeutic effects deserve more intensive investigation. The strategy of stem cells cultured with herbal extracts may be a feasible means which enhances the efficiency of stem cells and may be a useful and promising cell therapy for ALF.

## Data Availability

All data generated or analyzed during this study are included in this article. However, further details are available from the corresponding author on reasonable request.

## Supplementary Materials

Supplementary Materials Identification of human umbilical cord-mesenchymal stem cells (hUCMSCs). Figure S1: flow cytometry analysis of the surface markers in hUCMSCs. Figure S2: adipogenic, chondrogenic, and osteogenic differentiation of hUCMSCs. Figure S3: the rats ALT and AST results of different groups in tables. Figure S4: crizotinib can decrease the expression of c-Met receptor in hUCMSCs. Figure S5: the original image of western blot of experiments (Figure 4(c)).
