# Supplementary material for: In a Rat Model of Acute Liver Failure, Icaritin Improved the Therapeutic Effect of Mesenchymal Stem Cells by Activation of the Hepatocyte Growth Factor/c-Met Pathway
Source: Evid Based Complement Alternat Med. 2019 Nov 7;2019:4253846. doi: 10.1155/2019/4253846 (PMC6935441; doi:10.1155/2019/4253846)
Supplement: Supplementary Materials — Identification of human umbilical cord-mesenchymal stem cells (hUCMSCs). Figure S1: flow cytometry analysis of the surface markers in hUCMSCs. Figure S2: adipogenic, chondrogenic, and osteogenic differentiation of hUCMSCs. Figure S3: the rats ALT and AST results of different groups in tables. Figure S4: crizotinib can decrease the expression of c-Met receptor in hUCMSCs. Figure S5: the original image of western blot of experiments (Figure 4(c)). [file 4253846.f1.pdf]

1.Identification of human umbilical cord-mesenchymal stem cells (hucMSCs)

hucMSCs presented a homogeneous population of spindle fibroblast-like cells. They positively expressed CD73, CD90， CD105， HLA-ABC and negatively expressed IgG1 CD19,CD34, CD11b, CD45,and HLADR by flow cytometry (Fig. S1). Numerous lipid droplets were observed with Oil-Red-O staining in hucMSCs after incubation with the adipogenic supplementation for 14 days(Fig. S2A) and positive staining of Toluidine Blue were shown after chondrogenic induction(Fig. S2B).Alizarin Red staining performed 2 weeks after osteogenic induction, showing a red-colored mineralized matrix in treated cells(Fig. S2C).

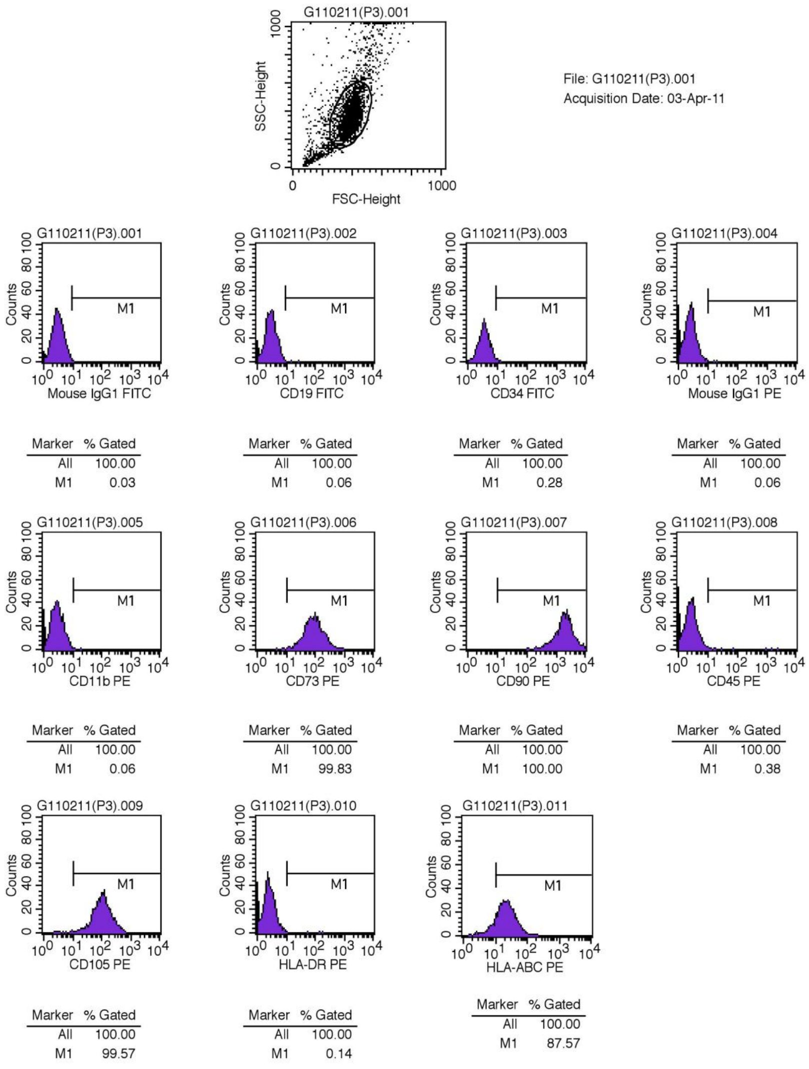

Fig. S1.Flow cytometry analysis of the surface markers in hucMSCs.

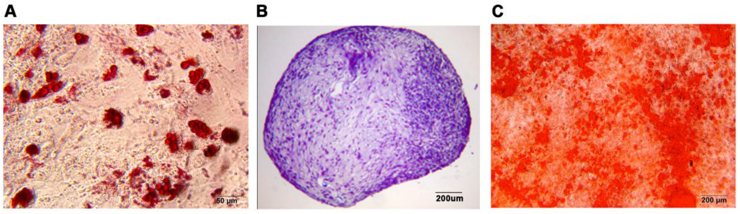

Fig. S2. Adipogenic、chondrogenic and osteogenic differentiation of hucMSCs (A) hucMSCs cultured in adipogenic medium. Results of Oil-Red-O staining detection in hucMSCs cultures grown for 14 days; (B) hucMSCs cultured in chondrogenic medium. Results of and staining detection with Toluidine Blue in hucMSCs cultures grown for 14 days (C)hucMSCs cultured in osteogenic medium. Results of Alizarin Red staining detection in hucMSCs cultures grown for 14 days.

2.AST and ALT enzyme levels in peripheral blood samples collected at 24 h and 48 h after model establishment(Fig. S3) .

Group	Time（H）	N	ALT(U/L)	AST(U/L)
NS	24	6	11579.83±2659.54	20366.50±2527.61
MSCs	24	6	8016.50±3290.90*	12287.83±5027.83*
ICT+MSCs	24	6	2584.50±1226.39$^{*\#}$	6058.16±2299.33$^{*\#}$
NS	48	4	11553.25±686.68	16978.75±1046.62
MSCs	48	6	3358.50±1605.91*	5766.00±2432.45*
ICT+MSCs	48	6	724.17±379.61$^{*\#}$	1717.50±989.59$^{*\#}$

Fig. S3.The rats ALT and AST results of different groups in tables, $^{*}P < 0.05$ *vs.* the NS group; $^{\#}P < 0.05$ *vs.* the MSCs group. Data are means ± SD. Abbreviations: MSCs, mesenchymal stem cells; ALT, alanine aminotransferase; AST, aspartate aminotransferase.

3.We used flow cytometry to examine the effect of c-Met receptor (crizotinib) on the expression of C-met receptor in human umbilical cord stem cells. It was found that the expression of the C-met receptor can be lowered by crizotinib. It confirmed that the c-Met receptor inhibition were successful.(Fig. S4)

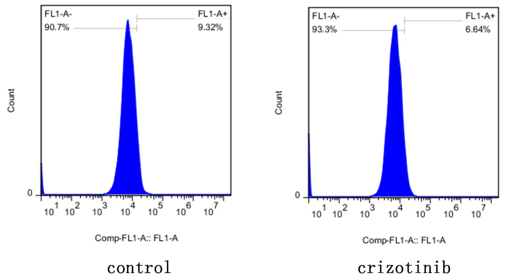

Fig. S4 Crizotinib can decrease the expression of C-met receptor inhucMSCs.

4.At the Reviewer 's Request,we provide the original image of western-blot of figure 4c to confirm the study results(Fig. S5).

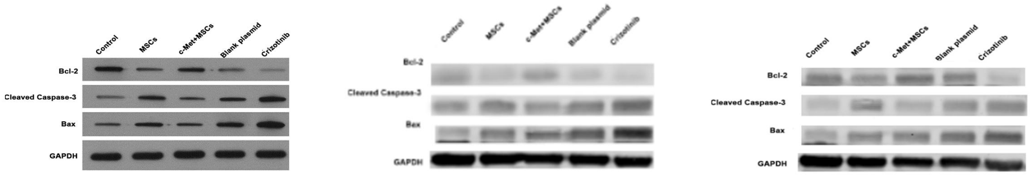

Fig. S5 The original image of western-blot of figure 4c（Since the setting conditions of electrophoresis and the film transfer are different, the two pictures on the right are different from the one on the left.）
